# Parental genetic diversity of brown trout (*Salmo trutta* m. *fario*) brood stock affects offspring susceptibility to whirling disease

**DOI:** 10.1186/s13071-015-0744-2

**Published:** 2015-03-03

**Authors:** Edit Eszterbauer, Barbara Forró, Zoltán Tolnai, Csaba Ferenc Guti, Gergely Zsigmond, György Hoitsy, Dennis Marc Kallert

**Affiliations:** Institute for Veterinary Medical Research, Centre for Agricultural Research, Hungarian Academy of Sciences, 21 Hungária krt, H-1143 Budapest, Hungary; Present address: Semmelweis University, Faculty of Medicine, 26 Üllői út, H-1085 Budapest, Hungary; Szent István University, Faculty of Veterinary Medicine, 2 István utca, H-1078 Budapest, Hungary; Lillafüred Trout Farm, 55 Erzsébet sétány, H-3517 Miskolc-Lillafüred, Hungary; Present address: Kallert & Loy GbR, 11 Birkenweg, 91325 Adelsdorf, Germany

**Keywords:** Individual inbreeding, Relatedness, Phylogeographic lineage, Brood fish, Microsatellite, *Myxobolus cerebralis*, Myxozoa

## Abstract

**Background:**

Whirling disease, caused by the myxozoan parasite *Myxobolus cerebralis*, has high economical and ecological importance worldwide. Susceptibility to the disease varies considerably among salmonid species. In brown trout (*Salmo trutta*) the infection is usually subclinical with low mortality, which increases the risk of parasite dissemination, especially when farm fish are used for stocking natural habitats. The influence of intraspecific genetic differences (especially the level of homozygosity) on susceptibility is unknown. Therefore, we examined the possible correlations between parental genetic diversity and offspring susceptibility of brown trout stocks to whirling disease.

**Methods:**

Two brown trout brood stocks from a German and a Hungarian fish farm were genetically characterized using microsatellite and lineage-specific genetic markers. The individual inbreeding coefficient *f* and pairwise relatedness factor *r* were estimated based on eight microsatellite markers. Brood stock populations were divided into groups according to low and high *f* and *r* value estimates and subjected to selective fertilization. The offspring from these separate groups were exposed to *M. cerebralis* actinospores, and the infection prevalence and intensity was measured and statistically analysed.

**Results:**

The analysis of phylogeographic lineage heritage revealed high heterogeneity in the Hungarian brood stock since > 50% of individuals were Atlantic-Danubian hybrids, while only pure Atlantic-descending specimens were detected in the German population. Based on *f*_msat_ and *r*_msat_ estimations, classified non-inbred (NIB), inbred (IB) and a group of closely related fish (REL) were created. The susceptibility of their offspring varied considerably. Although there was no significant difference in the prevalence of *M. cerebralis* infection, the mean intensity of infection differed significantly between NIB and IB groups. In REL and IB groups, a high variability was observed in infection intensity. No external clinical signs were observed in the exposed brown trout groups.

**Conclusions:**

Our findings indicate that the allelic diversity of brown trout brood stock may constitute a significant factor in disease susceptibility, i.e. the intensity of parasite infection in the subsequent generation.

## Background

The effects of inbreeding on salmonid reproduction, growth and survival have an extensive record in scientific literature. Numerous studies support the thesis of fitness-reducing negative effects, which may occur in fish with an increase in inbreeding [1-3]. The reasons for elevated inbreeding are usually the selection of less than optimal (and thus more closely related) individuals in artificial reproduction, the selection of certain desired phenotypes, the selection of spawning fish only during short sections of the spawning period, the use of only few female spawners due to sufficient amounts of eggs [4-6], the mixing of gametes of different brood stock animals [7,8], as well as differences in survival rates or sole re-use of certain parts of the brood stock population [9-12].

Besides these, selective breeding is often utilized for controlling economically relevant diseases. Through propagation of disease-survivors as brood stock, or the genetic manipulation of fish stocks, the disease resistance of fish populations can be increased [13-16]. In salmonids, resistant strains were developed against common diseases such as furunculosis [17] and viral haemorrhagic septicaemia (VHS) [18]. *Ceratonova* (syn *Ceratomyxa*) *shasta* was the first myxozoan parasite to which resistant strains were developed and evaluated [19,20]. In genetic studies on various rainbow trout (*Oncorhynchus mykiss*) strains with different susceptibility, several genome regions were found to be involved in resistance to *C. shasta*. The complex, multigenic trait of disease resistance has been confirmed for another myxozoan parasite, *Myxobolus cerebralis* [21-23]*.* The parasite responsible for whirling disease causes serious declines in wild and farmed salmonid populations worldwide. *M. cerebralis* infects a range of salmonid hosts that vary in susceptibility [24-27]. Fetherman *et al.* [28] showed that the introduction of a “*M. cerebralis*-resistant” rainbow trout strain to a natural habitat contributed to the reduction of infection prevalence and disease severity despite the low survival rate of stocked fish. Fish age and size also affect parasite development. The susceptibility of salmonid fry to the parasite decreases with age and growth [24,29-31]. Surprisingly, resistance is not associated with the level of skeletal ossification, but rather with other age- and size-related factors, such as the stage of development of the central nervous system [32]. Brown trout (*Salmo trutta*) is assumed to be the original host, which evolved with the parasite [33], and is considered one of the least susceptible salmonid host species. Infection in brown trout usually proceeds without clinical signs and modest to absent mortality. However, subclinical infections may increase the risk of the dissemination of the parasite into natural environments, especially in Europe, where hatchery-reared brown trout are used for stocking natural habitats, as a major part of conservation efforts [34]. Therefore, the role of reservoir hosts needs to be considered carefully and factored into management plans and epidemics as suggested for the monogenean ectoparasite *Gyrodactylus salaris* [35].

In the present study, we examined the correlations between parental genetic diversity and susceptibility traits of brown trout to the whirling disease parasite, *M. cerebralis*, for the first time. Marker heterozygosity-based grouping of brood stock was conducted on the basis of microsatellite and lineage-derived genetic markers, and the susceptibility of the offspring from assorted parental fish was evaluated in experimental infection trials in order to measure the effect of different homozygosity levels on the susceptibility of the resulting offspring.

## Methods

### Brood stock tagging and sampling

Fish were sampled in a Hungarian and a German trout hatchery in October, 2010, and in June, 2011. All examined fish were individually tagged with PIT tags (Loligo Systems). From the brown trout (BT) brood stock of Lillafüred trout hatchery in Hungary (48°6′59.22"N, 20°34′ 46.21"E), 167 specimens of 3–4 year-old fish were examined. From the brood stock of the trout hatchery in Aufseß, Germany (49°52′47.01"N, 11°13′41.43"E), 195 individuals (approx. equal number of males and females) from the same age group were sampled. During sampling, fin clips of approximately 1 cm^2^ were cut from the caudal fin of all specimens, photos were taken of every fish, and the total length was also recorded. Fin clips were fixed in 70% ethanol and stored at +4°C for molecular analysis.

### DNA extraction and lineage analysis

For DNA extraction, an approximately 5 mm^2^ fin clip cut was dried in a Speed Vac Concentrator (Savant), and homogenized in a 1.5 ml microcentrifuge tube with a sterile pestle (Eppendorf) in ultrapure MilliQ water. Extraction was carried out as described by Estoup *et al.* [36]. Briefly, 500 μl 10% Chelex 100 resin solution (BioRad) containing 0.3 mg/ml proteinase K was added to the homogenate and incubated under constant shaking at 55°C for 1 h. Digestion was terminated by heating to 100°C for 15 min. DNA extracts were stored at −20°C until further use. An approximately 1088 bp fragment of the mitochondrial DNA control region (mtDNA CR) was PCR-amplified using the primer pair RiBa (5’-CAC CCT TAA CTC CCA AAG CTA AG-3’) [37] and HN20 (5’-GTG TTA TGC TTT AGT TAA GC-3’) [38]. From nuclear DNA, a 428 bp fragment of the lactate-dehydrogenase gene C1 region (LDH-C1) was amplified with the primer pair Ldhxon3F (5’-GGC AGC CTC TTC CTC AAA ACG CCC AA-3’) and Ldhxon4R (5’-CAA CCT GCT CTC TCC CTC CTG CTG ACG AA-3’) [39]. The total volume of PCR reactions was 25 μl, which contained 15–40 ng DNA (0.5 μl), 1× *Taq* PCR reaction buffer (Fermentas, Thermo Scientific), 1.5 mM MgCl_2_, 0.2 mM dNTP mix (Sigma), 0.5 μM of each primer and 1.25 units of recombinant *Taq* DNA Polymerase (Fermentas, Thermo Scientific). For PCR conditions, the protocol described by Marić *et al.* [40] was followed.

The PCR-restriction fragment length polymorphism (PCR-RFLP) was conducted as per Marić *et al.* [40]. The amplified PCR products of mtDNA CR were digested with the restriction enzyme *Sat*I (Fermentas, Thermo Scientific), while *Bse*LI (Fermentas, Thermo Scientific) was used for LDH-C1 PCR products. The RFLP pattern was visualized on a 1.5% agarose gel in 0.5× TAE buffer stained with GelRed nucleic acid stain (Biotium). Photos were taken using a Gel Logic 212 Imaging System (KODAK).

### Microsatellite analysis

Eight microsatellite (msat) loci were examined on the tagged brood stock fish populations (Table 1). The DNA of msat Str-15, Str-60, Ssa-85 and Ssa-197 were amplified using a tetraplex PCR assay developed and optimized in the present study. The 25 μl total volume of PCR reaction contained approx. 50 ng template DNA, 1× AmpliTaq Gold PCR buffer (Life Technologies), 3 mM MgCl_2_, 40 μM dNTP (Sigma), 0.5 μM (for Str-15)/0.25 μM (for Ssa-197)/0.125 μM (for Ssa-85 and Str-60) of each primer and 1 u AmpliTaq Gold *Taq* DNA polymerase (Life Technologies). A duplex PCR to amplify msat Sso-197 and Str-543 was performed under similar conditions with 0.5 μM of each primer. Microsatellites SsoSL-438 and OKI-10 were amplified with simplex PCR assays containing half of the amount of MgCl_2_ and dNTP as mentioned above (1.5 mM and 20 μM, respectively). The PCR program started with an initial denaturation at 94°C for 5 min, followed by 35 cycles of 94°C/30 s, 55 or 60°C as indicated in Table 1 for 30 s and 72°C/30 s. The final elongation step was performed at 72°C for 3 min.

**Table 1:** **Oligonucleotides used for microsatellite analysis**

**Microsatellite locus**	**Oligonucleotide name**	**Sequence (5′- 3’)**	**Annealing temperature (°C)**	**PCR product size (bp)**	**Reference**
Str-15	Str15FAM	TGCAGGCAGACGGATCAGGC	60	220-226	[41]
	Str15R	AATCCTCTACGTAAGGGATTTGC			
Str-60	Str60FAM	CGGTGTGCTTGTCAGGTTTC	60	94-104	[41]
	Str60R	GTCAAGTCAGCAAGCCTCAC			
Str-543	Str543NED	ATTCTTCGGCTTTCTCTTGC	55	118-152	[42]
	Str543R	ATCTGGTCAGTTTCTTTATG			
SsoSL-417	SsoSL417HEX	TTGTTCAGTGTATATGTGTCCCAT	55	160-192	[43]
	SsoSL417R	GATCTTCACTGCCACCTTATGACC			
SsoSL-438	SsoSL438FAM	GACAACACACAACCAAGGCAC	55	98-108	[43]
	SsoSL438R	TTATGCTAGGTCTTTATGCATTGT			
Ssa-85	Ssa85NED	AGGTGGGTCCTCCAAGCTAC	60	104-116	[44]
	Ssa85R	ACCCGCTCCTCACTTAATC			
Ssa-197	Ssa197HEX	GGGTTGAGTAGGGAGGCTTG	60	128-158	[44]
	Ssa197R	TGGCAGGGATTTGACATAAC			
OKI-10	OKI10FAM	GGAGTGCTGGACAGATTGG	55	104-220	[45]
	OKI10R	CAGCTTTTTACAAATCCTCCT			

Forward primers were 5’end-labeled with fluorescent dyes FAM, NED and HEX, respectively. R: reverse primer.

The size of the msat loci were estimated with DNA fragment analysis on ABI 3100 Genetic Analyzer on POP6 polymer at 60°C using a GeneScan500 ROX size standard. Then, msat allele size detection was conducted with the software PeakScanner v1.0 (Life Technologies). Raw size values were normalized based on the length of the nucleotide (nt) repeats in the msat loci; e.g. for 2 nt repeats “CT” in Str-15, the size 199 was corrected to 200 as 1 nt difference is shorter than one 2 nt-repeat, therefore the two size values were treated identically (as they belong to the same number of 2-nt repeats).

Pairwise relatedness (*r*_msat_) and individual inbreeding coefficients (*f*_msat_) were estimated using the triadic likelihood estimator (TrioML) in the software COANCESTRY by J. Wang [46].

### Grouping of fish for selective fertilization

On the basis of the obtained data regarding the genetic status of BT brood stock, four groups were selected. Classified non-inbred (NIB) and inbred (IB) groups of parent fish in Aufseß were distinguished based on *f*_msat_ values. One group composed of a dozen NIB individuals of genetically related male and female parent fish (REL) was identified for individual fertilization based on *r*_msat_ values. Four male and female individuals from the Atlantic-Danubian hybrid (LBT) brood stock group were used for selective fertilization in the Lillafüred trout hatchery.

### Infection trial with *M. cerebralis*

Offspring of BT brood stock groups IB, NIB and REL were obtained from the trout hatchery in Aufseß, Germany, whereas LBT and Steelhead strain rainbow trout (*Oncorhynchus mykiss*; LRBT) fingerlings originated from the Lillafüred trout hatchery, Hungary (Table 2). Fish were kept in a parasite-free environment in both hatcheries before transport. Two month old fry were transported to the laboratory. They were kept in flow-through aquaria at 15 ± 2°C water temperature, and were fed with commercial trout food. The fish were exposed at the age of 3 months.

**Table 2:** **Fish groups used for infection trials with** ***Myxobolus cerebralis***

**Group identifier**	**Description**	**Species**	**Origin**
**NIB**	Non-inbred (heterozygous individuals)	Brown trout	Aufseß, Germany
**IB**	Inbred (homozygous individuals)	Brown trout	Aufseß, Germany
**REL**	Related (heterozygous, but related male–female pairs)	Brown trout	Aufseß, Germany
**LBT**	Atlantic-Danubian hybrid	Brown trout	Lillafüred, Hungary
**LRBT**	Positive control	Rainbow trout	Lillafüred, Hungary

Infective *Myxobolus cerebralis* triactinomyxon spores (TAMs) were obtained from our *in vivo* laboratory oligochaete cultures. TAMs were harvested by filtering the water from the culture containers through 20 μm mesh. TAMs used for the infection trials were less than 48 h old [47,48]. Fish were exposed individually to 3000 freshly filtered TAMs/fish in 500 ml dechlorinated tap water at 15°C for 3 h. Fifty-three individuals per group were exposed. Non-exposed control fish were kept under the same conditions with the exception of TAMs. After individual exposure, fish groups were kept separately in flow-through aquaria and fed daily. The infection experiment was terminated 4 months post exposure. Fish were euthanized with 200 mg.L^−1^ tricaine-methanesulfonate (MS222, Sigma), and kept frozen at −20°C until further examination.

Fish were decapitated and whole heads were divided horizontally. The cartilage and minced skeletal elements of a half head (including gill arches) were transferred into a 2 ml microcentrifuge tube containing a metal ball, and after the addition of 800 μl distillated water, the sample was homogenized with a TissueLyser LT (Qiagen) at 50 Hz for 10 min. Instead of quantifying parasite DNA of all developmental stages using species-specific PCR, morphologically intact *M. cerebralis* myxospores were counted which are transmission stages and play a key role in the propagation of the parasite. Spores were counted in 20 μl tissue homogenate on a microscopic slide with 20 × 20 mm coverslip under a light microscope (Zeiss Axiostar Plus) in three replicates.

### Statistical analysis

Statistical analyses were performed using the R program for Windows (R commander 2.15.1). A chi-square test was used for analyzing the difference in infection prevalence among BT groups. Difference of mean infection intensities among groups were tested by one-way ANOVA. Pairwise comparisons were made by Dunnett’s test with IB as the reference group. Non-infected individuals were excluded from the statistical analysis of infection intensity as suggested by Rózsa *et al.* [49].

### Ethical statement

We declare that the treatment of fish complied with the relevant Hungarian legislation (Section 49 of Act No. XXVIII/1998 on the protection and preservation of animals, and Executive decree No. 40/2013) and the Directive 2010/63/EU on the protection of animals used for scientific purposes and the guidelines and recommendations of the Federation of Laboratory Animal Science Associations.

## Results

### Lineage analysis

As a result of PCR-RFLP, one genotype was detected using mtDNA CR, and three genotypes with LDH-C1. For all examined samples, the PCR-RFLP pattern characteristic of the Atlantic (At) lineage was detected, as the amplified fragment of mtDNA CR was cut into two fragments, a 654 and a 434 bp long one. The pure Danubian (Da) genotype, whose amplified mtDNA CR fragment is not cut by the restriction endonuclease *Sat*I, was not present in the collected fish samples. In the German brown trout population, only the Atlantic allele was detected with the PCR-RFLP of LDH-C1. In every examined fish sample, the endonuclease *Bse*LI cut the 428 bp LDH-C1 fragment at one restriction site, resulting in 353 and 75 bp fragments. Thus, 100% of the examined fish from Germany belonged to the LDH-C1_At-At (Atlantic) genotype.

In the Hungarian brood stock population, both Atlantic (LDH-C1_At) and Danubian (LDH-C1_Da) LDH-C1 alleles were present, and the PCR-RFLP patterns of At (LDH-C1_At-At), Da (LDH-C1_Da-Da) and At-Da hybrid (Hyb) genotypes (LDH-C1_At-Da) were distinguishable (Table 3). Half of the Hungarian fish population showed the hybrid LDH-C1 pattern, while the genotype Da was detected in 25 cases of 144 (17%). All of the fish having LDH-C1_Da-Da (Da) showed the At pattern using mtDNA, which might be explained by back-crossing of hybrid parents. The gender difference was recognizable since more females than males (23% and 12.5% respectively) belonged to the Da genotype, which was the opposite for the hybrids (Table 3).

**Table 3:** **Frequency of mtDNA CR and LDH-C1 haplotypes/alleles and LDH-C1 genotypes of brood stock on the basis of PCR-RFLP pattern**

	**Female**		**Male**		**Total**	
	**Nr. of individuals**	**Frequency (%)**	**Nr. of individuals**	**Frequency (%)**	**Nr. of individuals**	**Frequency (%)**
**genetic markers**						
Da mtDNA	0/64	0	0/80	0	0/144	0
At mtDNA	64/64	100	80/80	100	144/144	100
LDH-C1_At	70/128	55	98/160	61	168/288	58
LDH-C1_Da	58/128	45	62/160	39	120/288	42
**LDH-C1 genotypes**						
LDH-C1_Da-Da (Da)	15/64	23	10/80	12.5	25/144	17
LDH-C1_At-At (At)	21/64	33	28/80	35	49/144	34
LDH-C1_At-Da (Hyb)	28/64	44	42/80	52.5	70/144	49

mtDNA CR: mitochondrial DNA control region; LDH-C1: lactate-dehydrogenase C1 region; Da: Danubian, At: Atlantic, Hyb: Atlantic-Danubian hybrid. The examined brown trout brood stock originated from the Lillafüred trout farm, Hungary.

### Microsatellite analysis

The size range of the examined msat loci is displayed in Table 1. The highest allele size variability was detected for OKI-10, for which 23 different msat length values have been observed in the examined brood stock. The slightest length variation was detected for Str-15 and Str-60 (4 different msat lengths each).

**Figure 1:**
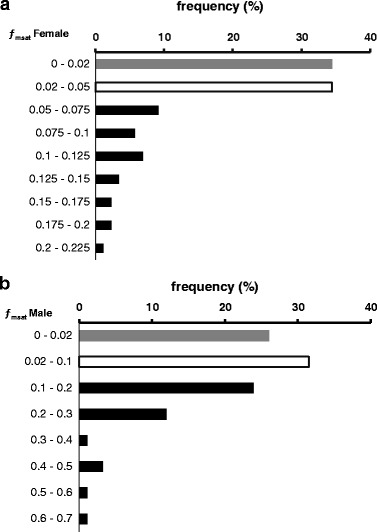
**Frequency of the microsatellite-based individual inbreeding coefficient (** ***f***_ msat_ **) values among the examined brown trout brood stock.** Data for female **(a)** and male **(b)** parents are displayed separately. The *f*_msat_ columns of non-inbred group are shown gray; those of the inbred group are black. Empty columns indicate the intermediate *f*_msat_ values excluded from the experiments.

The estimated *f*_msat_ values varied between 0 and 0.2091 for females, and 0 and 0.6197 for males. The examined brood stock specimens were divided into groups with relatively low and high individual inbreeding coefficients with thresholds resulting from the *f*_msat_ estimation. These were *f*_msat_ > 0.05 for more homozygous (IB) and *f*_msat_ < 0.02 for more heterozygous (NIB) females and *f*_msat_ > 0.1 for more homozygous (IB) and *f*_msat_ < 0.02 for more heterozygous (NIB) males. The latter threshold resulted from significantly higher individual *f*_msat_ values for NIB males, while fish with intermediate values were excluded from the experiments. The frequency distribution of *f*_msat_ values showed that while more than 40% of males could be classified as inbred based on our markers, only about 30% of females had *f*_msat_ values that made them candidates for the IB group (Figure 1).

The assignment of closely related males to selected females from the NIB group (all individuals’ *f*_msat_ < 0.02) for experimental mating of related and non-related parents (paired samples from single female egg batches) was based on *r*_msat_ estimation. Non-related parents had an *r*_msat_ < 0.0002, close-sib (highly related) pairs had a *r*_msat_ > 0.2. The *r*_msat_ values of male–female pairs considered related (REL) varied between 0.2208 and 0.4481.

The inbreeding coefficient *f*_msat_ of randomly selected NIB and IB offspring individuals (ten of each group) has also been estimated and showed high variance within the groups as expected (NIB: 0.0002 - 0.3414; IB: 0.0004 - 0.1353).

### Infection trial with *M. cerebralis*

**Figure 2:**
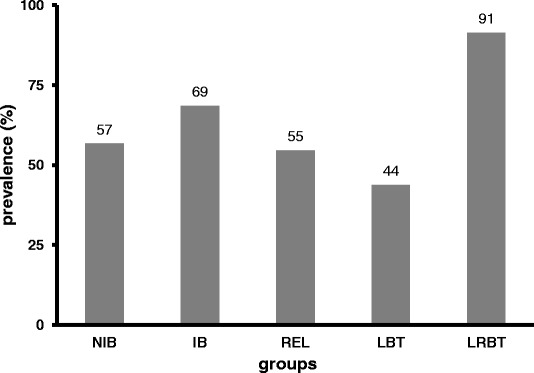
**Prevalence of** ***Myxobolus cerebralis*** **infection in experimentally exposed brown trout groups of different genetic status.** NIB: non-inbred, IB: inbred, REL: related, LBT: Atlantic-Danubian hybrid brown trout, and LRBT: rainbow trout (positive control).

**Figure 3:**
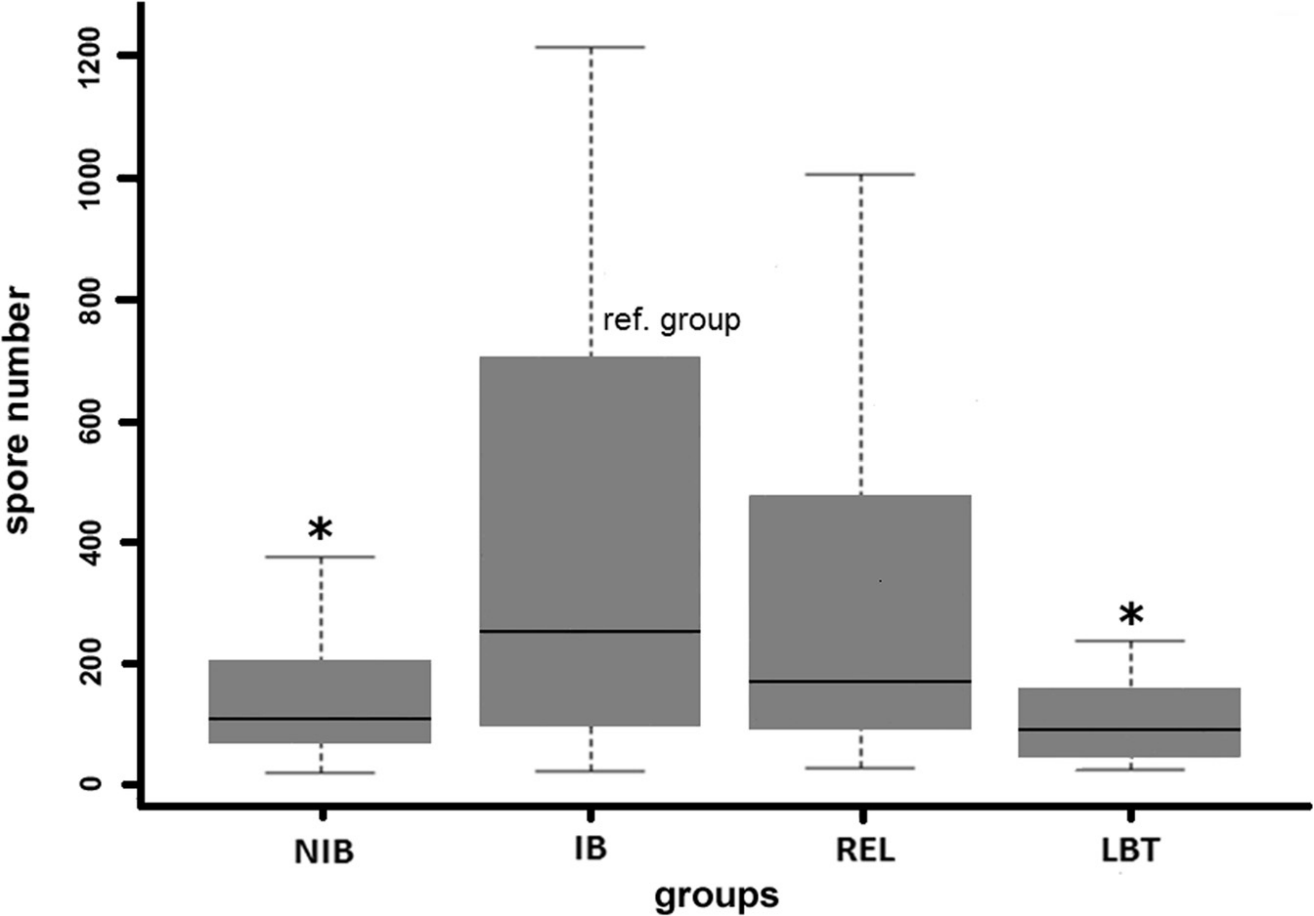
**Mean intensity of** ***Myxobolus cerebralis*** **infection in the examined brown trout groups.** NIB: non-inbred, IB: inbred, REL: related individuals, LBT: Atlantic-Danubian hybrid group. *: significant difference from the reference (inbred) group (P < 0.005). Non-infected individuals were excluded from the analysis.

The exposed BT groups showed no clinical signs of infection. The infection prevalence was highest (91%) in the positive control group (LRBT) with moderate clinical signs in the affected fish specimens (blackened tail, growth retardation and slight spinal deformations). In the non-exposed, negative control fish, *M. cerebralis* myxospores were not detected. The prevalence of infection varied between 44 and 69% among BT groups, but the difference was not significant between any of the BT groups (Figure 2). However, the intensity of *M. cerebralis* infection showed a more remarkable difference. The observed mean spore number was highest for the IB group (274 ± 344.54; mean spore number ± standard deviance, SD) and lowest for LBT (52 ± 85.41). The group NIB and REL were mid-ranged regarding mean spore numbers (91 ± 126.98 and 174 ± 274.72, respectively), while in the LRBT group an extreme variance in spore numbers was observed (8517 ± 25793.39). When comparing all values to that of the group IB as a reference, the infection intensity of the NIB and the At-Da hybrid group, LBT was significantly lower than that of the reference group (*p* < 0.005). The related group, REL did not differ significantly from the reference IB group (Figure 3).

## Discussion

Inbreeding reduces the rates of reproduction and survival in most regularly outbred species [50]. But inbreeding depression can not only lead to a strong reduction of growth in salmonids [51]. According to other studies, inbreeding causes increased embryo mortality, and affects spawning age and egg counts of female rainbow trout [4,52-54]. Interestingly, inbred males had no effect on hatching success, whereas inbreeding in female spawners had fatal consequences for developing eggs and caused poor hatching rates [46]. Our findings support these observations, as more IB females failed to ovulate in the spawning period than in the NIB group.

Until now, heterozygosity-fitness correlations remain in criticism. The subject of controversy is the question whether relationships between degree of homozygosity and observed fitness parameters are detectable and comprehensible at all. According to Balloux *et al.* [55] increased heterozygosity is a rather poor indicator for genome-wide inbreeding and also a large number of markers can reflect the inbreeding coefficient *f* only under extreme conditions of kinship mating. Even pedigree data, as preferred by most authors, computationally tend to underestimate the real degree of inbreeding. The decisive advantage of the marker-based approach is the possibility to investigate the existing inbreeding (−depression) in animals with identical origin (and thus same expected *f*) on the basis of individual values. To circumvent the mentioned problems, especially when using microsatellite data, Wang [46] proposed a new probability estimator, TrioML which is better correlated with fitness parameters and weighted for allelic diversity of a given population. Therefore, in the present study, the TrioML estimator was chosen for the calculation of *f*_msat_ to give a more reliable estimation of the homozygosity level of the examined brood stock.

The signs of whirling disease and their severity vary between fish host species. Among other species such as Atlantic salmon (*Salmo salar*), brown trout usually is an asymptomatic carrier of *M. cerebralis* and can act as a vector for the parasite [56]. Approximately 20 d post exposure, the parasite locates in the cartilage (primarily in the opercula, cranium and spine) causing lesions and inflammation, and potentially damaging the skeletal structure of the fish [57]. Locations of lesions vary among fish species and can explain variability in disease severity and survival among different species. Cartilage lesions, observed by histology in rainbow trout, tend to concentrate in the cranium around the brain, but can be found throughout the cartilage in the body in young fry [27]. However, in brown trout, lesions concentrate in fin rays and gill arches [58,59]. Under experimental conditions, Hedrick *et al.* [58] compared the relative susceptibility of age-matched brown and rainbow trout fry to *M. cerebralis*. Using different TAM doses (from 10 to 10000 TAMs/fish), they compared the effect of exposure dose on the prevalence of clinical signs, the severity of microscopic lesions, the presence and number of spores in the cartilage of infected fish, and the relative risk of infection for each fish species. In our study, a dose of 3000 TAMs/fish was used for exposure and a significant difference was detected both in prevalence and intensity of infection, similarly to the findings of Hedrick *et al.* [58] when using > 1000 TAMs/fish.

Although Nichols *et al.* [20] and Fetherman *et al.* [23] presumed that the geographic distribution of fish strains might have an influence on the genetic basis of resistance to both *C. shasta* and *M. cerebralis*, the correlation between the phylogeographic composition of salmonid populations and their resistance to the diseases has not been studied so far. Our study is also the first to compare the susceptibility of different genetic lineages of brown trout. The examined German brood stock represents a homogenous population, while the Hungarian population shows high heterogeneity concerning its phylogeographic lineages. Genetic analysis determined that the German population is Atlantic-descendant, while the Hungarian brood stock merely contains a higher ratio of At markers than the autochthonous Da one, and thereby > 50% of brood stock is comprised of At-Da hybrids. And the heterogenous group LBT composed of At and At-Da hybrid parent fish was the least susceptible to *M. cerebralis* of all examined fish groups.

As a key finding, the fully heterozygous group NIB was significantly less susceptible to the parasite than the more homozygous IB group. Thus, our findings suggest that there is a positive correlation between degree of parental heterozygosity and disease resistance. Higher variability in infection intensity was observed in the groups IB and REL compared to NIB and LBT, albeit the prevalence of infection did not differ significantly among the examined brown trout groups. The variations in groups IB and REL was mainly caused by a small fraction of specimens (<5%) that showed extremely heavy infections compared to their conspecifics. This was the case in the LRBT group as well, of course with even higher variance in spore numbers. Individual differences in resistance to certain myxozoan-caused diseases including whirling disease are well-known [60]. In most cases, the individual differences in immune competence or the development of the innate immune system are the causes of variations in disease resistance. But in the present study, individual variations in the susceptibility of offspring might be also caused by the allele recombination of parent fish pairs/groups that may result higher variations in the homozygosity level of the offspring population than in that of the parental one. The findings of the genetic analysis of randomly selected offspring individuals support this, as we showed that IB brood fish also had NIB offspring and vice versa. The brood stock fish in REL group were heterozygous, but genetically closely related individuals were paired, therefore the relatively high susceptibility of REL offspring can be explained by the “forced” inbreeding caused by the selective fertilization. The opposite overall tendency could be observed in the IB group, in which only the parents were inbred and the selective fertilization probably outbred the offspring, at least to a certain extent. In the offspring of IB and REL, not only the elevated homozygosity may be a reason for the increased parasite susceptibility, but the resulting allele depletion that lowers immune diversity and versatility. The variations in infection intensity were less remarkable in the offspring of the heterozygous groups NIB and LBT, probably because these brood stock fish were non-related and the heterozygosity level did not decrease considerably in offspring.

## Conclusions

Our findings indicate that the homozygosity level and thus the genetic diversity of brood stock has a significant influence on the intensity of parasite infection in the subsequent generations. As there is no effective treatment against whirling disease as yet, preventive measures against the parasite should be accompanied by regular genetic diversification of brood stock under controlled conditions to avoid increased levels of homozygosity in brood stock populations, even if barely measurable.

## References

[1] Charlesworth D, Charlesworth B (1987). Inbreeding depression and its evolutionary consequences. Ann Rev Ecol Syst.

[2] Falconer DS, Mackay TFC (1996). Introduction to Quantitative Genetics.

[3] Lynch M, Walsh B (1998). Genetics and Analysis of Quantitative Traits.

[4] Kincaid HL (1983). Inbreeding in fish populations used for aquaculture. Aquaculture.

[5] Flagg TA, Waknitz FW, Maynard DJ, Milner GB, Mahnken CVW (1995). The effect of hatcheries on native coho salmon populations in the lower Columbia River. Am Fish Soc Symp.

[6] Wang SZ, Hard J, Utter F (2002). Salmonid inbreeding: a review. Rev Fish Biol Fish.

[7] Tave D (1986). Genetics for Fish Hatchery Managers.

[8] Withler RE, Beacham TD (1994). Genetic consequences of the simultaneous or sequential addition of semen from multiple males during hatchery spawning of chinook salmon (*Oncorhynchus tshawytscha*). Aquaculture.

[9] Simon RC (1991). Management techniques to minimize the loss of genetic variability in hatchery fish populations. Am Fish Soc Symp.

[10] Kincaid HL (1995). An evaluation of inbreeding and effective population size in salmonid broodstocks in federal and state hatcheries. Am Fish Soc Symp.

[11] Geiger HA, Smoker WW, Zhivotovsky LA, Gharrett AJ (1997). Variability of family size and marine survival in pink salmon (*Oncorhynchus gorbuscha*) has implications for conservation biology and human use. Can J Fish Aquat Sc.

[12] Hard JJ, Connell L, Hershberger WK, Harrell LW (2000). Genetic variation in mortality of chinook salmon (*Oncorhynchus tshawytscha*) during a bloom of the marine alga *Heterosigma akashiwo*. J Fish Biol.

[13] Herman RL, Snieszko SF (1970). Prevention and control of fish diseases in hatcheries. Symposium on diseases of fisheries and shellfishes. Special Publication 5.

[14] Price DJ (1985). Genetics of susceptibility and resistance to disease in fishes. J Fish Biol.

[15] Chevassus B, Dorson M (1990). Genetics of resistance to disease in fishes. Aquaculture.

[16] Wiegertjes GF, Stet RJM, Parmentier HK, van Muiswinkel WB (1996). Immunogenetics of disease resistance in fish: a comparative approach. Dev Comp Immunol.

[17] Ehlinger NF (1977). Selective breeding of trout for resistance to furunculosis. N Y Fish Game J.

[18] Slierendrecht WJ, Olesen NJ, Juul-Madsen HR, Lorenzen N, Henryon M, Berg P (2001). Rainbow trout offspring with different resistance to viral haemorrhagic septicaemia. Fish Shellfish Immunol.

[19] Ibarra AM, Hedrick RP, Gall GAE (1994). Genetic analysis of rainbow trout susceptibility to the myxosporean *Ceratomyxa shasta*. Aquaculture.

[20] Nichols KM, Bartholomew J, Thorgaard GH (2003). Mapping multiple genetic loci associated with *Ceratomyxa shasta* resistance in *Oncorhynchus mykiss*. Dis Aquat Org.

[21] Schisler GJ, Myklebust KA, Hedrick RP (2006). Inheritance of *Myxobolus cerebralis* resistance among F1-generation crosses of whirling disease resistant and susceptible rainbow trout strains. J Aquat Anim Health.

[22] Baerwald MR, Petersen JL, Hedrick RP, Schisler GJ, May B (2011). A major effect quantitative trait locus for whirling disease resistance identified in rainbow trout (*Oncorhynchus mykiss*). Heredity.

[23] Fetherman ER, Winkelman DL, Schisler GJ, Antolin MF (2012). Genetic basis of differences in myxospore count between whirling disease-resistant and susceptible strains of rainbow trout. Dis Aquat Org.

[24] Halliday MM (1976). The biology of *Myxosoma cerebralis*: the causative organism of whirling disease of salmonids. J Fish Biol.

[25] Hoffman GL (1990). *Myxobolus cerebralis*, a worldwide cause of salmonid whirling disease. J Aquat Anim Health.

[26] Hedrick RP, El-Matbouli M, Adkison MA, MacConnell E (1998). Whirling disease: re-emergence among wild trout. Immunol Rev.

[27] MacConnell E, Vincent ER, Bartholomew JL, Wilson JC (2002). The effects of *Myxobolus cerebralis* on the salmonid host. Whirling disease: Reviews and current topics. American Fisheries Society, Symposium 29.

[28] Fetherman ER, Winkelman DL, Baerwald MR, Schisler GJ (2014). Survival and Reproduction of *Myxobolus cerebralis*-Resistant Rainbow Trout Introduced to the Colorado River and Increased Resistance of Age-0 Progeny. PLoS One.

[29] Markiw ME (1991). Whirling disease: Earliest susceptible age of rainbow trout to the triactinomyxid of *Myxobolus cerebralis*. Aquaculture.

[30] Markiw ME (1992). Experimentally induced whirling disease. I. Dose response of fry and adults of rainbow trout exposed to the triactinomyxon stage of *Myxobolus cerebralis*. J Aquat Anim Health.

[31] El-Matbouli M, Fischer-Scherl T, Hoffmann RW (1992). Present knowledge of the life cycle, taxonomy, pathology, and therapy of some Myxosporea spp. important for freshwater fish. Ann Rev Fish Dis.

[32] Ryce EKN, Zale AV, MacConnell E, Nelson M (2005). Effects of fish age versus size on the development of whirling disease in rainbow trout. Dis Aquat Org.

[33] Hoffman GL, Snieszko SF (1970). Intercontinental and transcontinental dissemination and transfaunation of fish parasites with emphasis on whirling disease (*Myxosoma cerebralis*). Symposium on diseases of fisheries and shellfishes. Special Publication 5.

[34] Laikre L, Antunes A, Apostolidis A, Berrebi P, Duguid A, Ferguson A (1999). Conservation genetic management of brown trout (*Salmo trutta*) in Europe. Report by the concerted action on identification, management and exploitation of genetic resources in the brown trout (*Salmo trutta*).

[35] Paladini G, Hansen H, Williams CF, Taylor NGH, Rubio-Mejía OL, Denholm SJ (2014). Reservoir hosts for *Gyrodactylus salaris* may play a more significant role in epidemics than previously thought. Parasites Vectors.

[36] Estoup A, Largiader CR, Perrot E, Chourrout D (1996). Rapid one-tube DNA extraction for reliable PCR detection of fish polymorphic markers and transgenes. Mol Marine Biol Biotech.

[37] Snoj A, Jug T, Melkic E, Susnik S, Pohar J, Dovc P (2000). Mitochondrial and microsatellite DNA analysis of marble trout in Slovenia. J Freshwater Biol (Quaderni ETP).

[38] Bernatchez L (2001). The evolutionary history of brown trout *Salmo trutta* L. inferred from phylogeographic, nested clade, and mismatch analyses of mitochondrial DNA variation. Evolution.

[39] McMeel OM, Hoey EM, Ferguson A (2001). Partial nucleotide sequences, and routine typing by polymerase chain reaction-restriction fragment length polymorphism, of the brown trout (*Salmo trutta*) lactate dehydrogenase, LDH-C1-C1*90 and *100 alleles. Mol Ecol.

[40] Marić S, Simonović P, Razpet A (2010). Genetic characterization of brood stock brown trout from Bled fish-farm, Slovenia. Period Biologorum.

[41] Estoup A, Presa P, Krieg F, Vaiman D, Guyomard R (1993). CT)n and (GT)n microsatellites: a new class of genetic markers for *Salmo trutta* L. (brown trout. Heredity.

[42] Presa P, Guyomard R (1996). Conservation of microsatellites in three species of salmonids. J Fish Biol.

[43] Slettan A, Olsaker I, Lie Ø (1995). Atlantic salmon, *Salmo salar*, microsatellites at the SSOSL25, SSOSL85, SSOSL311, SSOSL417 loci. Anim Gen.

[44] O’Reilly PT, Hamilton LC, McConnell SK, Wright JM (1996). Rapid analysis of genetic variation in Atlantic salmon (*Salmo salar*) by PCR multiplexing of dinucleotide and tetranucleotide microsatellites. Can J Fisheries Aquat Sci.

[45] Smith CT, Koop BF, Nelson RJ (1998). Isolation and characterization of coho salmon (*Oncorhynchus kisutch*) microsatellites and their use in other salmonids. Mol Ecol.

[46] Wang J (2011). COANCESTRY: A program for simulating, estimating and analysing relatedness and inbreeding coefficients. Mol Ecol Resources.

[47] Kallert DM, El-Matbouli M (2008). Differences in viability and reactivity of actinospores of three myxozoan species upon ageing. Folia Parasitol.

[48] Eszterbauer E, Kallert DM, Grabner D, El-Matbouli M (2009). Differentially expressed parasite genes involved in host recognition and invasion of the triactinomyxon stage of *Myxobolus cerebralis* (Myxozoa). Parasitology.

[49] Rózsa L, Reiczigel J, Majoros G (2000). Quantifying parasites in samples of hosts. J Parasitol.

[50] Keller LF, Waller DM (2002). Inbreeding effects in wild populations. Trends Ecol Evol.

[51] McKay LR, McMillan I, Sadler SE, Moccia RD (1992). Effects of mating system on inbreeding levels and selection response in Salmonid aquaculture. Aquaculture.

[52] Aulstad D, Gjedrem T, Skjervold H (1972). Genetic and environmental sources of variation in length and weight of rainbow trout (*Salmo gairdneri*). J Fish Res Board Canada.

[53] Gjerde B, Gunnes K, Gjedrem T (1983). Effect of inbreeding on survival and growth in rainbow trout. Aquaculture.

[54] Su GS, Liljedahl LE, Gall GAE (1996). Effects of inbreeding on growth and reproductive traits in rainbow trout (*Oncorhynchus mykiss*). Aquaculture.

[55] Balloux F, Amos W, Coulson T (2004). Does heterozygosity estimate inbreeding in real populations?. Mol Ecol.

[56] Steinbach Elwell LC, Stromberg KE, Ryce EKN, Bartholomew JL (2009). Whirling Disease in the United States. A summary of progress in research and management 2009.

[57] El-Matbouli M, Hoffmann RW, Mandok C (1995). Light and electron microscopic observations on the route of the triactinomyxon-sporoplasm of *Myxobolus cerebralis* from epidermis into rainbow trout cartilage. J Fish Biol.

[58] Hedrick RP, McDowell TS, Gay M, Marty GD, Georgiadis MP, MacConnell E (1999). Comparative susceptibility of rainbow trout *Onchorhynchus mykiss* and brown trout *Salmo trutta* to *Myxobolus cerebralis*, the cause of salmonid whirling disease. Dis Aquat Org.

[59] Baldwin TJ, Vincent ER, Silflow RM, Stanek D (2000). *Myxobolus cerebralis* infection in rainbow trout (*Oncorhynchus mykiss*) and brown trout (*Salmo trutta*) exposed under natural stream conditions. J Vet Diag Invest.

[60] Gómez D, Bartholomew J, Sunyer JO (2014). Biology and mucosal immunity to myxozoans. Dev Comp Immunol.

